# Sinonasal Melanoma: A Case Report and Literature Review

**DOI:** 10.1155/2017/8201301

**Published:** 2017-01-31

**Authors:** I. S. S. Alves, L. G. S. Berriel, R. T. Alves, M. B. Pinto, C. F. P. Oliveira, A. C. Cazzotto, W. V. Moura

**Affiliations:** ^1^Hospital Universitário Cassiano Antônio de Moraes, Santos Dumont, ES, Brazil; ^2^Hospital Santa Casa de Misericórdia de Vitória, Vitória, ES, Brazil

## Abstract

*Purpose*. Sinonasal malignant mucosal melanoma is a rare, aggressive tumour. Nasal obstruction and epistaxis are the most commonly reported symptoms, although symptomatology may develop late and be nonspecific, which tends to delay diagnosis, resulting in a poorer prognosis.* Case Report*. This report describes a 64-year-old male patient with nasal obstruction and epistaxis. Computed tomography of the facial sinuses revealed a large lesion in the right nasal cavity, with infiltration into the left cavity, ethmoidal cells, and erosion of the cribriform plate. Initial incisional biopsy revealed an undifferentiated carcinoma of the right maxillary sinus, staged as T4aN0M0. Induction chemotherapy was initiated with cisplatin and etoposide. Response to treatment was complete. The patient was then submitted to radiotherapy with concomitant cisplatin. Immunochemical analysis revealed positivity for vimentin, S100, and HMB-45 (human melanoma black 45), a result compatible with a diagnosis of malignant melanoma.* Discussion*. Due to the rarity of the tumour and the patient's complete response to chemotherapy and since no blackened lesion had been found at the previous exam, treatment was continued as planned. The patient remains healthy, with no metastasis or recurrence. He is currently being monitored by the clinical oncology team.

## 1. Introduction

First described by Lucke in 1869 [1], sinonasal malignant melanoma is a rare, aggressive tumour associated with poor prognosis. It accounts for 0.5–2% of all melanomas [2–8]. Its incidence is slightly higher in men, in whites, and in over 60s [4, 9]. Risk factors and pathogenesis remain to be clarified [10]. Symptoms develop slowly; therefore, many patients are diagnosed late, reducing overall survival [9]. The risk of local recurrence (31–85%) and distant metastasis (25–50%) is high [11, 12]. Diagnosis is based on anatomopathological and immunohistochemical findings [13]. The treatment of choice is surgical resection, while radiotherapy and chemotherapy serve to control local and metastatic disease [14].

This report on a case of malignant mucosal melanoma includes a brief literature review. The internal review board of the* Irmandade da Santa Casa de Misericórdia de Vitória* approved this report on June 28, 2016, under reference number 56846116.9.0000.5065. The patient gave his written informed consent.

## 2. Case Report

A 64-year-old white male consulted at a public healthcare facility in Mantena, Minas Gerais, Brazil, in February 2013 with pain on the right side of his face and nose. He reported sporadic episodes of epistaxis and progressive ipsilateral nasal obstruction beginning about a year previously.

Computed tomography (CT) of the facial sinuses revealed a large, solid, voluminous mass in the right nasal cavity, measuring 3.8 × 3.1 × 5.1 cm along its largest axes and extending into the left nasal cavity, ethmoidal cells, and cribriform plate, causing erosion of the plate. Incisional biopsy was performed and histology revealed an undifferentiated carcinoma of the right maxillary sinus.

The patient was referred to the* Santa Casa de Misericórdia* Hospital in Vitória, Espírito Santo, Brazil. Since surgical resection proved impossible, the condition was treated clinically. Symptomatology persisted and diplopia developed. The patient's general health was good, with no asthenia, anorexia, or weight loss. Karnofsky index was 90%. Induction chemotherapy consisted of three cycles of etoposide and cisplatin and was followed by six cycles of cisplatin concomitantly with radiotherapy.

**Figure 1:**
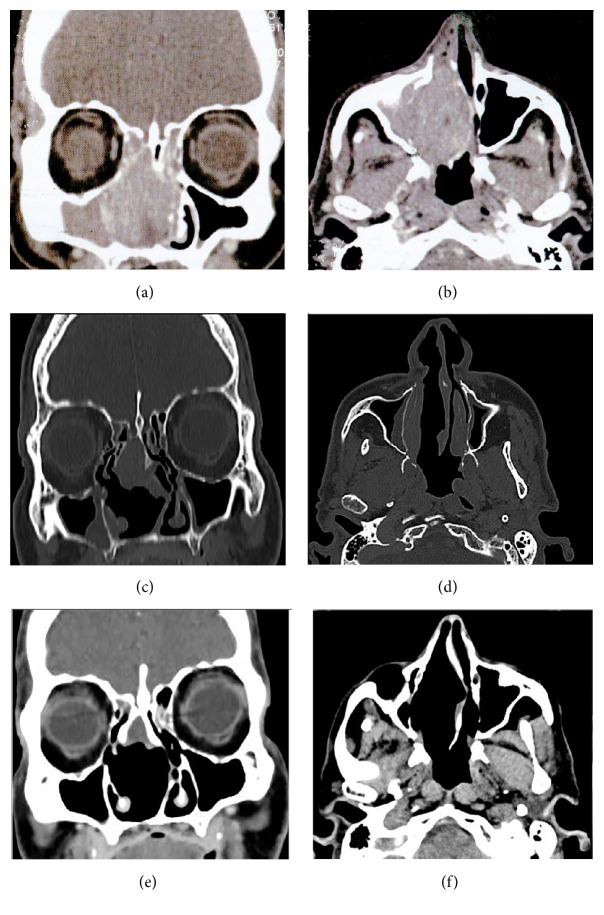
(a) and (b): pretreatment: computed tomography of the facial sinuses. Presence of a solid voluminous mass extending throughout the right nasal cavity, measuring 3.8 × 3.1 × 5.1 cm along its longest axes. This mass is infiltrating the left nasal cavity, ethmoidal cells, and cribriform plate, causing erosion to the plate. (a) Coronal plane. (b) Axial plane. (c) and (d): midway through treatment: computed tomography of the facial sinuses. (c) Presence of a solid lesion affecting the ethmoidal cells, nasal cavity, and right sphenoid sinus, with the tumour extending as far as the floor of the sella turcica on the right, with erosion. No signs of pituitary involvement. Widening of the sphenoethmoidal recess due to infiltration of the tumour mass. Thickening of the mucosa occupying the maxillary sinuses bilaterally, principally on the right (coronal plane). (d) Lesion lodged in the right nasal cavity with deviation of the septum to the left (axial plane). (e) and (f): following treatment: computed tomography of the facial sinuses. Improvement of the sphenoid and right maxillary sinuses, with almost complete resolution of the lesions seen in previous exams. (e) Coronal plane. (f) Axial plane.

**Figure 2:**
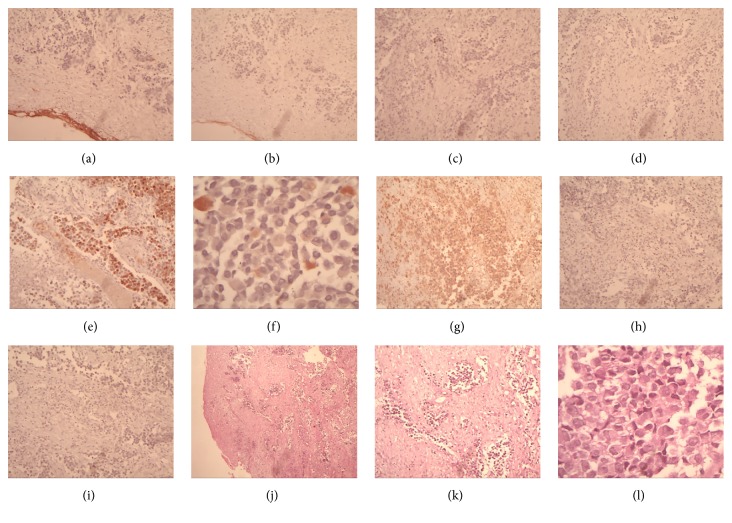
Immunohistochemical profile and histology of the lesion. (a) AE1 + AE3. (b) CD 138. (c) CK 8/18. (d) Desmin. (e) HMB 45. (f) S100 (×40)—focal positivity. (g) Vimentin (×20)—diffuse. (h) CD3. (i) CD20. (j) HE ×10. (k) HE ×20. (l) HE ×40. Pleomorphic, polyhedral giant cells with large hyperchromatic nucleoli with a diffuse pattern.

The histopathological report was reviewed, revealing an undifferentiated ulcerated neoplasm. CT of the brain and paranasal sinuses showed a solid lesion affecting the ethmoidal cells, nasal cavity, and right sphenoid sinus and extending as far as the floor of the sella turcica on the right side, causing erosion; there were no signs of pituitary involvement. Infiltration into the sphenoethmoidal recess causes widening. Thickening of the mucosal tissue occupied the maxillary sinuses bilaterally, predominantly on the right. The lesions were lodged in the right nasal cavity, deviating the septum to the left. CT of the neck showed no lymph node involvement. The patient was staged as cT4aN0M0 [15]. Immunohistochemistry showed positivity for vimentin, S100, and HMB45 (human melanoma black 45), compatible with malignant melanoma (see Figures 1 and 2 and the following).

*TNM Staging in Cases of Sinonasal Melanoma*

*Primary Tumour (T)*

T*x*: the primary tumour cannot be evaluated.
T3: disease restricted to the mucosa.
T4a: disease moderately advanced: tumour deeply involving soft tissue, cartilage, bone, or overlying skin.
T4b: very advanced disease: tumour involving brain, dura mater, skull base, cranial nerves (IX, X, XI, and XII), masticator space, carotid artery, prevertebral space, or the mediastinal structure.

*Regional Lymph Nodes (N)*

N*x*: the regional lymph nodes cannot be evaluated.
N0: there is no evidence of regional nodal metastases.
N1: presence of regional nodal metastases.

*Distant Metastases (M)*

M0: no distant metastases.
M1: distant metastases.

In view of the rarity of the tumour, the patient's complete response to induction chemotherapy, and the fact that no blackened lesion had been found at the previous exam, treatment proceeded as planned, even after diagnosis was changed during chemotherapy. Over the 21 months of follow-up with CT scans every 3 months, there were no signs of recurrence or metastasis and an improvement was seen in the patient's right sphenoid and maxillary sinus, with almost complete resolution of the lesions detected in previous exams. The patient remains healthy and is being followed up.

## 3. Discussion

Primary mucosal sinonasal melanoma corresponds to 0.5–2% of all malignant melanomas and approximately 4% of melanomas of the head and neck [2–7]. Mean 5-year survival is 0–30% [16]. The incidence of malignant mucosal melanoma is lower in areas where the incidence of malignant skin melanoma is high. According to the American College of Surgeons, of the 84,836 melanomas seen over a 9-year period, only 1.3% developed on mucosal surfaces. Of these, 55% were on the head and neck [6]. The principal differences between malignant skin melanoma and malignant mucosal melanoma are listed in Table 1 [17–19].

The condition predominantly affects individuals ≥60 years of age. Although possible at any age, it is extremely rare in young people [20] and slightly more common in males [21–23]. The most common sites are the nasal cavity, septum, inferior and middle nasal conchae, the lateral wall of the nasal cavity, and the facial sinuses [24]. The paranasal sinuses are rarely affected but, of these, the maxillary sinus is the most commonly affected [22].

Etiopathogenesis remains unclear; however, mucosal melanomas are known to develop from melanocytes present in mucosal tissue originating from the migration of cells from the neural crest during the embryonic period [25–29].

No specific risk factors are known. The long-term presence of melanosis is the association that has been most clearly demonstrated in the case of oral melanoma [30]. Occupational exposure to formaldehyde has been classified as a possible risk factor [23, 29, 31]. In this particular patient, no obvious risk factor was present.

The absence of symptoms in the initial stages delays diagnosis. Furthermore, it is often difficult to differentiate primary melanoma from metastases [32]. The most common initial symptoms are unilateral nasal obstruction and epistaxis. Associated symptoms include rhinorrhea, hyposmia, frontal headache, facial pain, proptosis, diplopia, and epiphora [13]. The prognosis of patients with epistaxis may be better than that of those with nasal obstruction alone [33]. This patient had the classic symptoms of this type of tumour—epistaxis and nasal obstruction—and was diagnosed at an advanced stage.

Anterior rhinoscopy and fibre optic nasolaryngoscopy may reveal blackish-blue, pale yellow, or, in the case of amelanotic melanomas, translucent polypoid masses [34]. The primary tumour site is often difficult to determine due to the extension of the lesions at diagnosis. In this case, various structures were affected simultaneously; however, the principal site was the right maxillary sinus, thus characterizing the primary site of the lesion. When satellite lesions are present and there are concomitant areas of amelanotic melanoma, the case becomes more complicated [1]. The fact that the melanoma here was amelanotic demanded a much more detailed differential diagnosis.

Neck nodal metastasis occurs in 10–50% of patients [13, 35, 36]. Distant metastases occur in 40–76% of patients and may affect the lung, liver, brain, skin, and orbit [37, 38]. In patients affected by mucosal melanomas, the possibility of skin metastases demands careful dermatological examination [13]. In this case, there were no nodal or distant metastases, either at diagnosis or follow-up. Tomography and magnetic resonance imaging supply sufficient data to determine staging, location, and regional extension and to rule out the existence of lesions involving the meninges, brain tissue, and major vascular structures [32].

Definitive diagnosis is based on immunohistochemistry. Great care should be taken with diagnosis if immunohistochemistry is not performed [13]. In the present case, the initial diagnosis was based on histopathology alone, which led to a diagnostic error that was only corrected following immunohistochemistry. Due to the difficulty in diagnosing a malignant sinonasal melanoma, differential diagnoses must be made with sinonasal undifferentiated carcinoma, lymphoma, rhabdomyosarcoma, angiosarcoma, neuroendocrine carcinoma, neuroblastoma, and plasmacytoma [29].

Most mucosal melanomas are of the lentiginous, superficial, or nodular types [39, 40]. Histologically, they consist of large fusiform or epithelioid cells with abundant eosinophilic cytoplasm. Around one-third of tumours are undifferentiated and can be confused with other types of tumour. The immunohistochemical profile is identical to that of a skin melanoma. Markers include S-100 protein, HMB-45, melan-A, microphthalmia-associated transcription factor, tyrosinase, vimentin, and cytokeratin. Positivity for S-100 is not always present; therefore, another set of markers must be investigated to establish diagnosis. Since there was positivity for S-100 and HMB-45 in the present case, malignant melanoma was diagnosed. Certain histopathological characteristics of this type of tumour are predictive of poorer survival, including vascular invasion, necrosis, and a polymorphous population of tumour cells. Characteristics such as the thickness of the tumour, the degree of invasion, ulceration, mitotic index, and neural involvement appear to have little effect on prognosis, showing that the relevant histological characteristics for prognosis in cases of malignant mucosal melanoma differ significantly from those of malignant skin melanomas [41, 42].

First-line treatment consists of surgical resection [43]. Prophylactic neck nodal dissection at N0 is not recommended, since the incidence of occult nodal metastases is relatively low [41]. Local recurrence occurs in 29–79% of cases despite aggressive surgery and may be clinically devastating [44–49]. Some authors recommend radiotherapy for local control [50, 51], while others have reported no improvement in overall survival [52]. Chemotherapy, most commonly with actinomycin D, interferon, and cisplatin, is used at more advanced stages, generally for palliative purposes or when surgery is contraindicated [9, 53]. In addition to interferon [54], interleukin 2 and bacillus Calmette-Guérin are other possible options for immunotherapy [55]. Here, chemotherapy and neoadjuvant radiotherapy were given, and the patient responded well. Surgery may be an option later.

For patients with sinonasal melanoma, prognosis is poor, since this tumour is aggressive and normally diagnosed at more advanced stages. More than 50% of patients die within three years of diagnosis [1], some within a few weeks or months of developing symptoms, with the disease disseminating rapidly despite surgical treatment [53]. Here, the disease was advanced at diagnosis; however, the structures of the central nervous system were not affected. Contradicting global statistics, this patient remains healthy, with no signs of metastasis or recurrence.

## Figures and Tables

**Table 1:** Comparison between malignant skin melanoma and mucosal melanoma.

Characteristics	Skin	Mucosal
Tissue of origin	Skin	Mucosal surface
Mean age [17]	50 years	60 years
Clinical condition	<1/3 with advanced disease	>50% with advanced disease
Amelanotic appearance	1.8–8.1% [6]	20–25% [18, 19]
Risk factor	Sun exposure	Unknown
Ethnic group	White: 94%	White: 85%
	Black: 0.8%	Black: 7%
Adjuvant radiotherapy	Not recommended	Local control
